# Adipose-derived stem cells attenuate pulmonary microvascular hyperpermeability after smoke inhalation

**DOI:** 10.1371/journal.pone.0185937

**Published:** 2017-10-05

**Authors:** Koji Ihara, Satoshi Fukuda, Baigalmaa Enkhtaivan, Raul Trujillo, Dannelys Perez-Bello, Christina Nelson, Anita Randolph, Suzan Alharbi, Hira Hanif, David Herndon, Donald Prough, Perenlei Enkhbaatar

**Affiliations:** 1 Department of Anesthesiology, The University of Texas Medical Branch, Galveston, Texas, United States of America; 2 Department of Plastic and Reconstructive Surgery, Kagoshima City Hospital, Kagoshima, Japan; 3 Department of Plastic and Reconstructive Surgery, Tokyo Women’s Medical School, Tokyo, Japan; 4 Department of Surgery, Shriners Hospital for Children, Galveston, Texas, United States of America; National Yang-Ming University, TAIWAN

## Abstract

**Background:**

Pulmonary edema is a hallmark of acute respiratory distress syndrome (ARDS). Smoke inhalation causes ARDS, thus significantly increasing the mortality of burn patients. Adipose-derived stem cells (ASCs) exert potent anti-inflammatory properties. The goal of the present study was to test the safety and ecfficacy of ASCs, in a well-characterized clinically relevant ovine model of ARDS.

**Methods:**

Female sheep were surgically prepared. ARDS was induced by cooled cotton smoke inhalation. Following injury, sheep were ventilated, resuscitated with lactated Ringer’s solution, and cardiopulmonary hemodynamics were monitored for 48 hours in a conscious state. Pulmonary microvascular hyper-permeability was assessed by measuring lung lymph flow, extravascular lung water content, protein content in plasma and lung lymph fluid. Sheep were randomly allocated to two groups: 1) ASCs: infused with 200 million of ASCs in 200mL of PlasmaLyteA starting 1 hours post-injury, n = 5; 2) control, treated with 200mL of PlasmaLyteA in a similar pattern, n = 5.

**Results:**

Lung lymph flow increased 9-fold in control sheep as compared to baseline. Protein in the plasma was significantly decreased, while it was increased in the lung lymph. The treatment with ASCs significantly attenuated these changes. Treatment with ASCs almost led to the reversal of increased pulmonary vascular permeability and lung water content. Pulmonary gas exchange was significantly improved by ASCs. Infusion of the ASCs did not negatively affect pulmonary artery pressure and other hemodynamic variables.

**Conclusions:**

ASCs infusion was well tolerated. The results suggest that intravenous ASCs modulate pulmonary microvascular hyper-permeability and prevent the onset of ARDS in our experimental model.

## Introduction

Acute respiratory distress syndrome (ARDS) is a severe form of acute lung injury caused by sepsis, pneumonia, trauma, severe burn, and smoke inhalation injury [1]. Although survival from ARDS has increased in recent years with the use of intensive supportive cares, such as lung-protective ventilation and fluid-conservative management, the mortality of ARDS patients is still high [1]. Due to the lack of specific treatment, smoke inhalation injury and ARDS are a major cause of morbidity and mortality in burn patients [2]. Pathophysiological changes in the lungs after smoke inhalation injury are characterized by increased pulmonary microvascular permeability, edema formation, and airway obstruction. Chemical components of smoke stimulate the release of neuropeptides from peripheral endings of sensory neurons within the airways to induce neurogenic inflammation. Plasma extravasation and oedema then result as secondary responses. Neurogenic inflammation results in narrowing of airway lumina, which is attributable to airway mucosal hyperaemia, formation of obstructive casts in the airway, and bronchospasm. These changes result in severe impairment of respiratory gas exchange [3].

Mesenchymal stem cells (MSCs) have been shown to be beneficial in many pathological conditions, such as myocardial infarction [4], graft versus host disease [5] and spinal cord injury [6]. Believed to be multipotent cells, MSCs are capable of differentiating into multi cell types i.e., adipocytes, chondrocytes and osteocytes [7]. In addition, they can also differentiate into a variety of cell lineages that form mesenchymal tissues, such as marrow stroma, muscle, cartilage, tendon, fat, and bone [8–10]. Other studies have shown that MSCs lead to improved clearance of alveolar fluid and have anti-inflammatory effects on host tissue in preclinical models of ARDS and sepsis [11]. To date, two clinical studies (Phase 1) on the safety of MSCs use in patients with ARDS have been successfully completed [12, 13], and recently, we have reported on the beneficial effects of clinical grade human bone marrow-derived MSCs in ovine models of ARDS induced by pneumonia/sepsis [14].

In the present study, we tested the hypothesis that intravenously administered adipose-derived stem cells (ASCs) effectively ameliorate the severity of pulmonary microvascular hyper-permeability in ovine models of ARDS induced by smoke inhalation.

## Material and methods

### Animal care and use

This study was approved by the Institutional Animal Care and Use Committee of the University of Texas Medical Branch (1308034) and conducted in compliance with the guidelines of the National Institutes of Health and the American Physiological Society for the care and use of laboratory animals.

### Surgical preparation

Ten female Merino sheep weighing 30–40 kg were surgically prepared 5–7 days before the experiment. Under aseptic conditions, the animals were chronically instrumented with multiple vascular catheters for hemodynamic monitoring as previously described [15]. In brief, under isoflurane anesthesia (IsoSol, VEDCO, St. Joseph, MO) administered via endotracheal tube, a 7F Swan-Ganz thermodilution catheter (model 131F7, Edwards Critical Care Division, Irvine, CA) was inserted into the right jugular vein through a 9.0F Intro-Flex-Percutaneous Sheath Introducer (CENTURION, Williamston, MI) and was advanced into the common pulmonary artery. The right femoral artery was cannulated, and a polyvinylchloride catheter (16-gauge, 24-in., Intracath, Becton Dickinson Vascular Access, Sandy, UT) was positioned in the descending aorta. Through a left thoracotomy at the level of the fifth intercostal space, a Silastic catheter (0.062-in. inner diameter, 0.125-in. outer diameter; Dow-Corning, Midland, MI) was positioned in the left atrium. To determine pulmonary transvascular fluid flux (lung lymph flow), a thoracotomy in the fifth and seventh intercostal space was performed, and the efferent vessel of the caudal mediastinal lymph node was cannulated with Silastic medical grade tubing (0.025-in inner diameter, Dow Corning, Midland, MI) with a modified method based on the technique of Staub et al [16]. The animal was given a 5-day recovery period. During this time, they had free access to food and water. Pre and post surgical analgesia was provided with buprenorphine (Buprenorphine SR^™^, SR Veterinary Technologies, Windsor, CO).

### Measured variables

Before beginning studies, catheters were connected to pressure transducers (model PX4X4, Baxter Edwards Critical Care Division, Irvine, CA) with continuous flushing devices. Electronically calculated mean pressures (MAP: mean arterial pressure; CVP: central venous pressure; MPAP: mean pulmonary artery pressure; and LAP: left atrium pressure) were recorded on a monitor with graphic and digital displays (MP30, Philips, Andover, MA). Pressures were measured while sheep were standing and calm. Zero calibrations were taken at the olecranon joint on the frontal leg while the animals were standing. Core body temperature was measured with the thermistor of the Swan-Ganz catheter. 10mL of Saline solution at 1°C served as the thermal indicator. Arterial blood gas samples were analyzed at 37°C and carboxyhemoglobin (COHb) was measured using a conventional blood gas analyzer (RAPIDPoint 500 System, Siemens Healthcare Diagnostics, Tarrytown, NY); consequently corrected for core body temperature. The partial arterial oxygen pressure (PaO_2_)/inspired oxygen fraction (FiO_2_) ratio, cardiac index (CI), systemic vascular resistance index (SVRI), and oxygenation index (OI) were calculated using standard formulas. Lung lymph flow was measured with graduated test tubes and a stopwatch. Plasma and lymph protein concentrations were measured using a refractometer.

### Experimental protocol

After baseline (BL) measurements and sample collections were completed in the healthy state, a tracheostomy was performed under ketamine (KetaVed, Phoenix Scientific, St.Joseph, MO) anesthesia (5mg/kg) and a cuffed tracheostomy tube (10mm diameter, Shiley, Irvine, CA) was inserted into the trachea. In addition, a Foley urinary retention catheter (C.R. Bard, Inc., Covington, GA) was placed in the urinary bladder to monitor fluid balance. Anesthesia was maintained with 2% to 5%. Isoflurane (IsoSol, VEDCO, St. Joseph, MO) in O_2_. Smoke inhalation was induced using a modified bee smoker. The bee smoker was filled with 40 g of burning cotton towels and then attached to the tracheostomy tube via a modified endotracheal tube containing an indwelling thermistor from a Swan-Ganz catheter. Four sets of twelve breaths of smoke (total 48 breaths) were delivered, and the carboxyhemoglobin level was determined immediately after each set. The temperature of the smoke was not allowed to exceed 40°C during the smoking procedure [15, 17, 18]. Immediately after injury, anesthesia was discontinued, and the animals were allowed to awaken but were maintained on mechanical ventilation (Hamilton-G5, Hamilton Medical, Switzerland) throughout the 48-h experimental period.

After the injury, sheep were randomly allocated to one of the two groups (n = 5 each): 1) control (injured, treated with vehicle); and 2) ASCs treatment (injured, treated with 200 million of adipose-derived stem cells). The cells were administered intravenously over 30 min via central line, starting 1 h after injury.

All sheep were continuously (around the clock) monitored for 48 hours in the Translational Intensive Care Unit. The variables of hemodynamics, pulmonary function, blood gas exchange, and lung lymph flow (transvascular fluid flux) were recorded every 6 hours.

All sheep were mechanically ventilated in APVcmv mode with positive end-expiratory pressure set at 5 cmH_2_O, tidal volume maintained at 12mL/kg and a respiratory rate of 20 breaths per minute. The breath rate was periodically adjusted to maintain arterial carbon dioxide tension close to baseline values. One hundred percent oxygen was delivered in the first three hours after injury to accelerate the dissociation of carbon monoxide from hemoglobin. The fraction of inspiratory oxygen was periodically adjusted to maintain the arterial oxygen tension above 95 mmHg.

All sheep received fluid resuscitation during the experiment with lactated Ringer’s　solution following the Parkland formula. During experimental periods, the animals were allowed free access to food, but not to water to accurately measure fluid intake.

### ASCs isolation and culture conditions

Subcutaneous adipose tissue was isolated from healthy sheep from the left fifth intercostal incision in preparative surgery and was washed extensively with PBS containing 2% penicillin/streptomycin. The tissue was then minced (2mm or less). Each 10 grams of minced fat tissue was placed in processing 50 ml tube with 2.5 ml of Matrase^™^ and up to 30 ml of lactated Ringer’s solution. Fat tissue was incubated at 37°C for 2 hours using ARC System (InGeneron, Inc). Because ovine adipose tissue has higher levels of saturated fat compared to adipose tissue from humans, longer incubation time was needed for the enzymatic digestion of the fat. Following complete digestion, solution was filtered in Steriflip^™^ to collect the filtrate. The filtrate was concentrated using ARC System by centrifuge at 600g for 10 minutes. Two washing steps with 60 ml of lactated Ringer’s solution were applied in order to have a pellet of stromal vascular fraction. Finally, the pellet was re-suspended in complete media [Dulbelco’s Minimum Essential Medium with 10% FBS and 2% antibacterial/antimycotic solution (10,000IU/mL Penicillin; 10,000ug/mL Streptomycin)]. The final pellet was seeded into tissue culture flask of 175 cm^2^, and placed in the 5% CO_2_ and 37°C incubator. After incubating for 24 hours, the media was replaced to remove unattached cells and debris. Cells were cultured and frozen down in aliquots. Second passage cells were used for the experiments.

**Fig 1:**
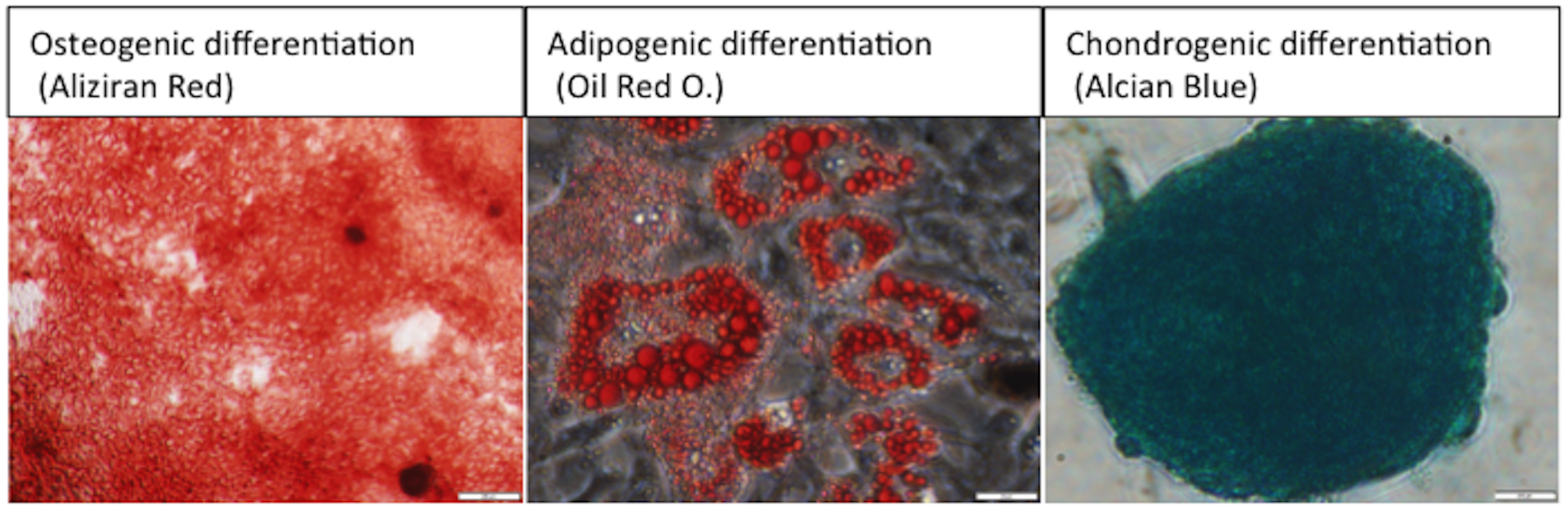
Multipotent differentiation potential of ASCs. The cells were differentiated into osteoblasts (demonstrated by staining with Alizarin Red), adipocytes (demonstrated by staining with Oil O Red) and chondroblasts (demonstrated by staining with Alcian blue).

The cells were characterizes as below: 1) adherence to plastic in standard culture conditions, 2) expression of specific surface antigen assessed by flow cytometer or PCR (Negative for CD45, CD31, CD14, CD11b and MHC Class II DQ/DR. Positive for CD44, CD73, CD90 and CD105), 3) differentiation potential for osteoblasts, adipocytes and chondroblasts (Fig 1).

### Necropsy

Forty-eight hours after injury, animals were deeply anesthetized and euthanized by intravenous administration of xylazine (3.0mg/kg), ketamine (40mg/kg), and buprenorphine (0.01mg/kg) following IACUC approved protocols, and American Veterinary Medical Association Guidelines for Euthanasia. The lower one-half of the lower lobe of the right lung was used for the determination of bloodless wet-to-dry weight ratio. The organ tissue samples were harvested, snap-frozen in liquid nitrogen and stored at -80°C for later analysis.

### Immunohistochemistry

For the distribution study, we prepared 3 sheep in the same manner, and treated with 200 million of GFP-labeled ASCs. Sheep were euthanized 24 hours after the injury. Several organs including the lung, liver, kidney, and spleen were removed, processed and paraffin embedded. 5 μm coronal sections were stained for histopathology analysis of ASCs distribution. In preparation for immunohistochemistry (IHC), samples were deparaffinized and rehydrated in xylene, 100% and 95% alcohol. Antigen retrieval was completed in 95°C 0.01M citrate buffer pH 6 followed by a quenching process of 3% endogenous hydrogen peroxide. The sections were blocked with the endogenous avidin- biotin complex (Life Technologies; Waltham, MA) to reduce nonspecific binding. Slides were stained with primary antibody rabbit anti- GFP (1:1000; Abcam Cambridge, MA) and secondary biotinylated anti- rabbit IgG (Vector Laboratories, Burlingame, CA). Horseradish peroxidase streptavidin (Vector Laboratories, Burlingame, CA) was used for color visualization in addition to diaminobenzidine (DAB; Dako, Carpinteria, CA). Sections were counterstained with Harris hematoxylin (Protocol, ThermoFischer Scientific, Waltham, MA).

### Statistical analysis

All values were expressed as means ± SEM. Statistic analysis (Prism 6 software [GraphPad Software, Inc. San Diego, CA]) was performed among the groups by two-way ANOVA, followed by post hoc Bonferroni test. COHb levels after smoke inhalation injury and wet-to-dry weight ratio between the groups were compared using non-parametric procedures (Mann-Whitney U test) after confirming non-normal distribution. A value of P < 0.05 was regarded as statistically significant.

## Results

All animals survived the 48-hour experimental period. The arterial carboxyhemoglobin levels determined immediately after smoke exposure averaged 74.6±4.3% in the control group and 69.5±3.3% in the ASCs group. There was no significant difference between these values.

**Fig 2:**
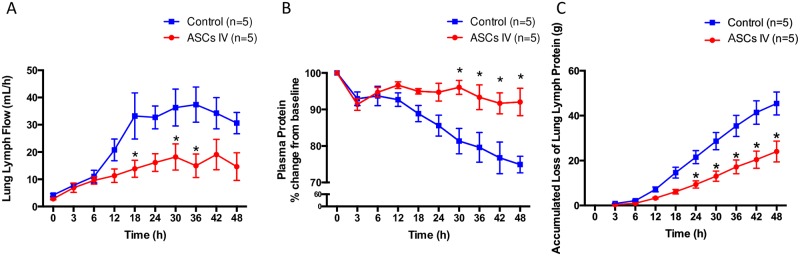
Fluid and protein leakage from lung. Intravenous administration of ASCs significantly reduced the pulmonary microvascular hyperpermeability in sheep caused by cotton smoke inhalation. (A) Lung lymph flow, an index of pulmonary transvascular fluid flux, was increased ~9-fold in control sheep compared to baseline. This was associated with a significant total protein decrease in plasma (B) and its increase in lung lymph(C). Treatment with ASCs prevented these changes.

**Fig 3:**
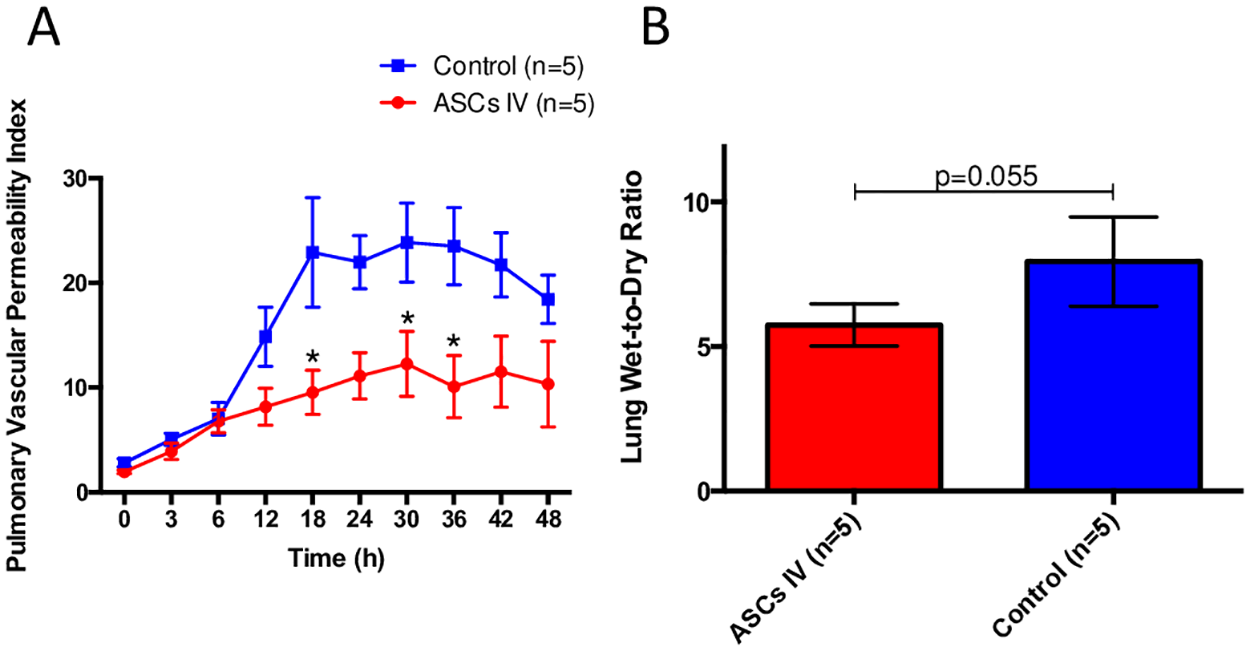
Pulmonary vascular permeability. (A) The treatment with ASCs almost reversed increased pulmonary vascular permeability index and (B) reduced lung water content (Lung Wet-to-Dry Ratio). Smoke inhalation injury causes pulmonary vascular hyperpermeability to both fluid and protein, which was attenuated by ASCs treatment.

Lung lymph flow, an index of pulmonary transvascular fluid flux, was increased ~9-fold in control sheep compared to baseline (37.4±14.4 at 36hr, 4.2±1.1 at Baseline). This was associated with a significant total protein decrease in plasma and its increase in lung lymph. The treatment with ASCs significantly attenuated increases in lymph flow (control vs. ASCs; 31.2±6.2 and 13.9±3.1 at 18hr, 33.3±5.6 and 18.2±4.8 at 30hr, 34.1±5.5 and 15.0±4.3 at 36hr, p<0.05) (Fig 2A). The plasma protein significantly decreased immediately after the injury vs. baseline and continued to drop in the control group. Treatment with ASCs prevented these changes (Fig 2B). Accumulated loss of protein with lung lymph tended to increase in both groups, but it was significantly lower after 24hrs in ASC group (Fig 2C). Moreover, the treatment with ASCs almost reversed increased pulmonary vascular permeability index {(lung lymph protein × lung lymph flow)/plasma protein} (Fig 3A) and reduced lung water content (Lung wet-to-dry weight ratio) (5.7±2.6 and 7.9±3.6, p = 0.0556) (Fig 3B).

**Fig 4:**
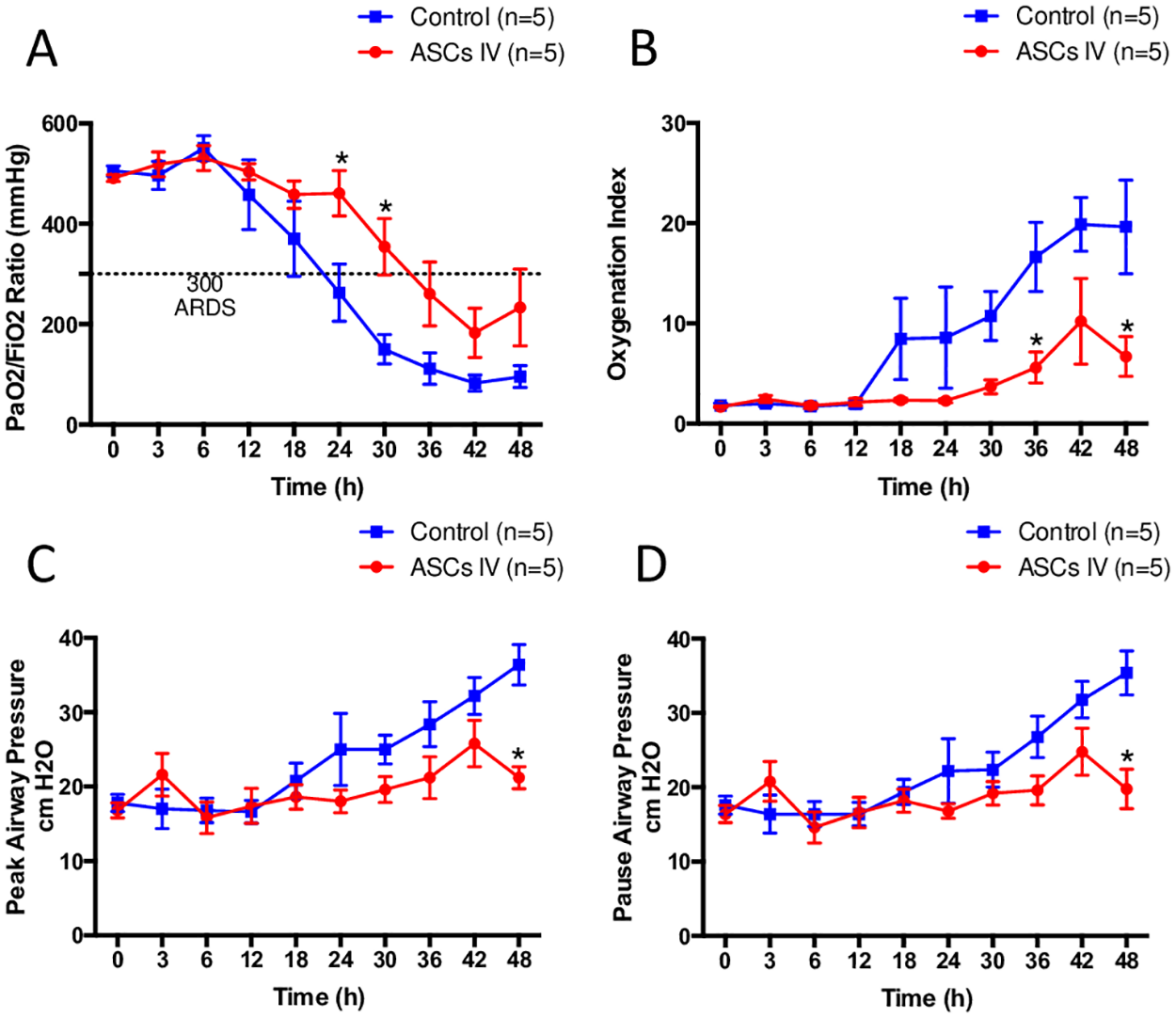
Pulmonary function. The administration of ASCs significantly improved pulmonary gas exchange, evaluated by determining PaO_2_/FiO_2_ ratio (A), and pulmonary oxygenation index (B). Peak and Pause airway pressures were gradually elevated after 18 hours and reached more than 2 fold in the control group at the end of the study. The treatment also significantly reduced elevated airway pressures (C, D).

PaO_2_/FiO_2_ ratio decreased below 300 after 24hours in control group. However, pulmonary gas exchange was significantly improved by ASCs treatment. ASCs treatment delayed the onset of ARDS (PaO_2_/FiO_2_ ratio, control vs. ASCs: 262±127 and 406±101 at 24hr, 150±29 and 354±56 at 30hr, p<0.05) (Fig 4A). Oxygenation index increased markedly in the control group after 18hr and continued to increase over the 48hrs study period. The latter was significantly attenuated by ASCs (P<0.05 at 36hr and 48hr [Fig 4B]).

Peak and Pause airway pressures were increased more than 2 fold in the control group. These changes were significantly inhibited by the ASCs treatment (Fig 4C and 4D).

**Fig 5:**
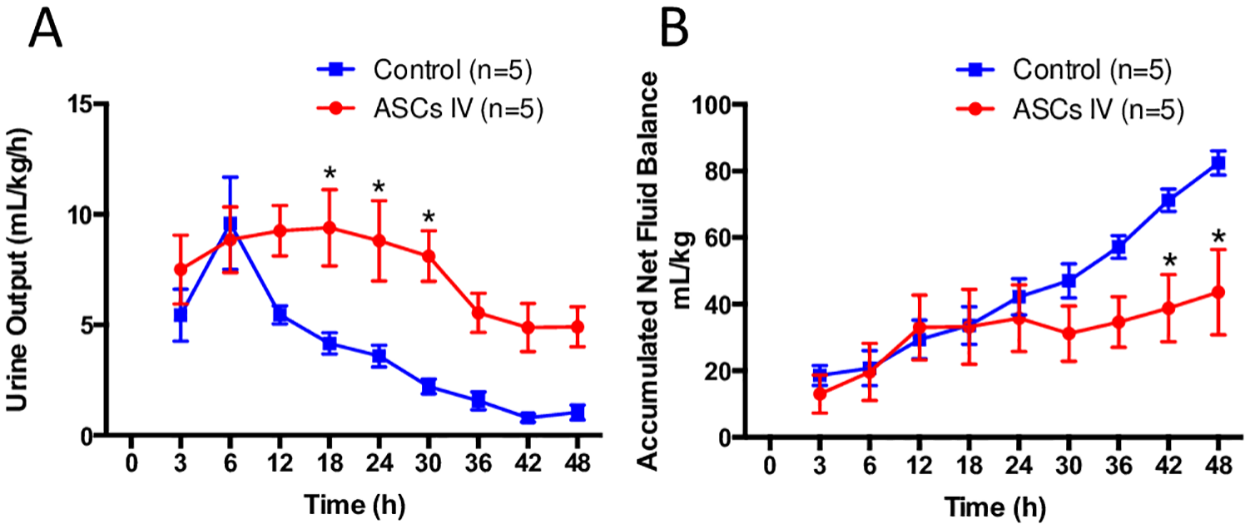
Fluid balance. (A) Urine output was significantly higher in the treatment group. (B) Increases in accumulated net fluid balance (fluid retention) were significantly attenuated by ASCs treatment compared to control group.

In treatment group, urine output was significantly higher compared to the control group at 18, 24 and 30hrs after injury (Fig 5A). Increases in accumulated net fluid balance (fluid retention) were significantly attenuated by ASCs treatment compared to control group at 42 and 48hrs after injury (Fig 5B).

### Systemic hemodynamics (Table 1)

**Table 1:** Systemic hemodynamics.

	Baseline	Time After Injury, h								
		3	6	12	18	24	30	36	42	48
MAP, mmHg										
Control	97.8±3.6	108±3.8	105±4.7	99±1.8	94.8±2.4	99.2±2.9	95.6±2.2	94.4±3.9	97.2±1.7	93±4.2
ASCs	96.6±3.1	114.4±3.4	111.0±3.0	103.8±3.2	101.8±2.6	96.8±4.0	97.6±1.5	99.0±4.0	104.4±2.1	101.8±3.0
PAP, mmHg										
Control	20.6±0.9	22.0±1.6	24.0±1.4	20.2±0.7	21.4±1.2	29.0±2.5	29.2±2.2	26.4±2.3	26.2±1.8	25.8±1.8
ASCs	22.2±2.4	27.4±1.7	28.2±1.3	26.4±2.8	30.4±2.7*	27.4±1.5	27,2±1.6	27.8±1.9	28.4±1.4	26.2±2.3
PCWP, mmHg										
Control	12.4±0.9	13.2±1.7	14.0±1.6	10.6±0.4	11.4±0.5	15.8±0.7	15.6±2.1	14.0±2.1	14.0±1.8	14.2±1.8
ASCs	13.2±1.9	17.0±1.2	15.2±0.6	14.4±1.2	16.0±1.7	15.2±0.7	15.0±1.2	14.8±2.0	14.6±1.9	15.0±1.8
CVP, mmHg										
Control	8.2±1.0	7.4±1.2	7.6±1.0	6.6±1.0	6.2±1.0	9.0±1.3	6.0±1.6	7.6±1.9	10.2±1.6	10.8±1.7
ASCs	7.8±1.4	11.0±1.2	10.4±0.7	7.8±0.9	9.4±0.8	9.8±1.6	9.0±1.7	8.0±2.0	9.0±2.1	9.4±2.2
CI, L×min^-1^×m^-2^										
Control	6.1±0.4	6.2±0.5	5.9±0.2	5.6±0.3	5.4±0.2	5.1±0.6	5.1±0.4	5.2±0.3	5.6±0.5	5.9±0.2
ASCs	6.4±0.3	6.7±0.4	6.5±0.4	6.3±0.2	6.1±0.2	6.2±0.5	5.3±0.2	6.0±0.4	6.8±0.7	6.0±0.7
SVRI, dynes×sec×cm^-5^m^-2^										
Control	1204.2±100.7	1322.4±94.0	1325.5±66.9	1336.7±75.8	1329.1±54.3	1457.9±132.9	1444.3±117.3	1356.1±69.6	1275.3±116.6	1123.2±60.2
ASCs	1120.9±44.0	1250.9±98.9	1262.7±105.5	1226.8±19.3	1208.2±59.3	1153.3±95.0	1348.2±87.5	1219.0±68.7	1177.7±142.4	1314.5±180.6

(MAP, mean arterial pressure; CVP, central venous pressure; MPAP, mean pulmonary artery pressure; and LAP, left atrium pressure)

Mean arterial pressure increased in both groups following injury as compared to baseline values. There was no difference in comparison between the two groups. Systemic vascular resistance also similarly increased post-injury in both groups. Additionally, the pulmonary capillary wedge pressure and central venous pressure did not deviate between the two groups over the experimental period.

The pulmonary artery pressure was not affected during the 30-minute infusion of 200 million ASCs into the jugular vein. In the post-injury time, the changes in pulmonary artery pressure were comparable in both groups except it was significantly higher at 18hrs in ASCs group. Cardiac index displayed no sustained post-injury changes, and there were no differences between sheep receiving therapeutic treatment and control group.

### Histopathology

**Fig 6:**
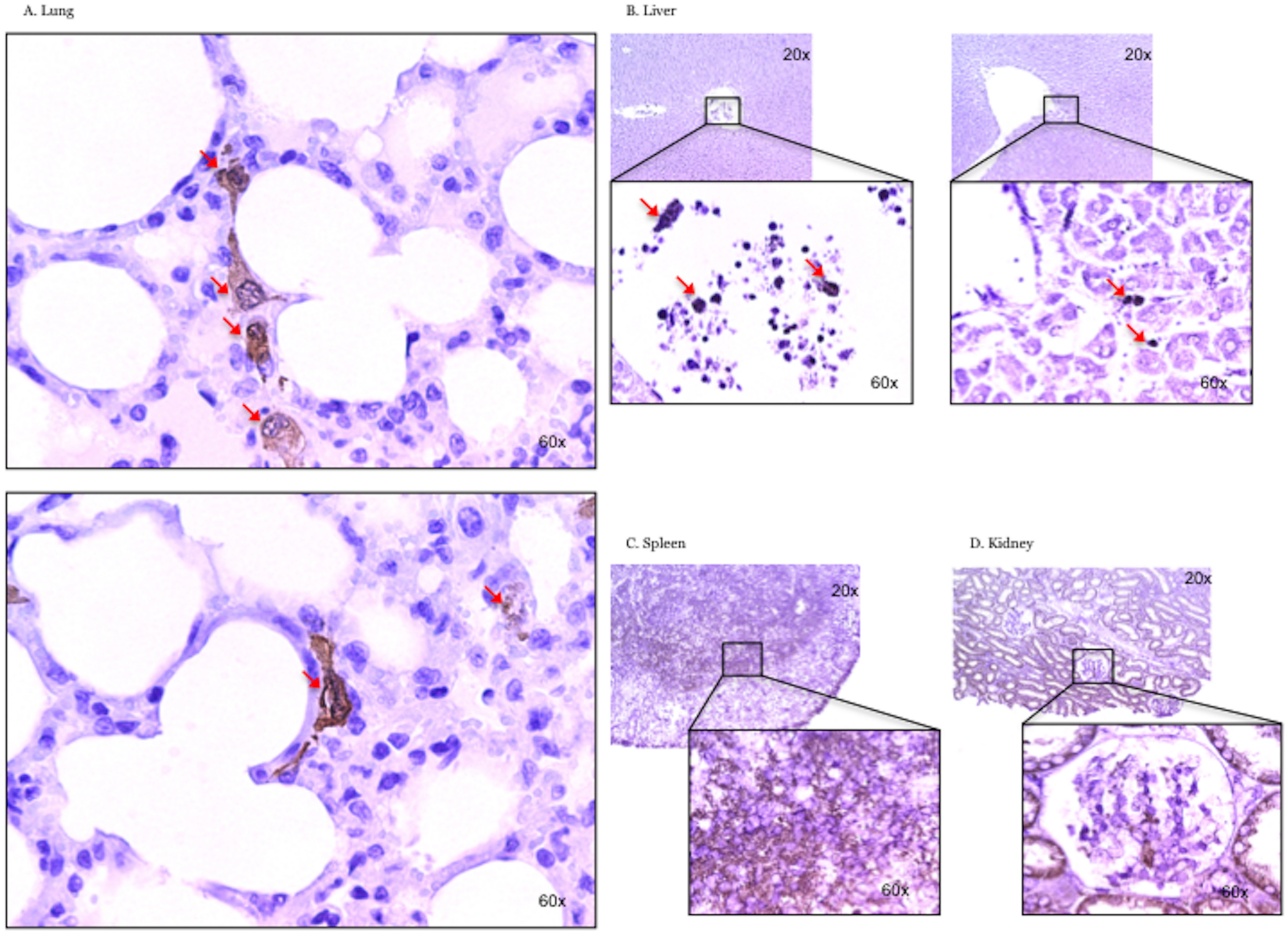
Histopathology. Many structurally intact ASCs were detected in the lung interstitial space (A). Few stained cells were found in liver (B). In the spleen, many smaller particles were stained by anti-GFP antibody (C). Urinary tubules were filled by stained particles (D).

Many structurally intact ASCs were detected in the lung interstitial space and seemed to migrate by changing their shape (Fig 6A). Few stained cells were found in liver vasculatures and sinus 24hours after injury (Fig 6B). In spleen, many smaller particles were stained by anti-GFP antibody, and no structurally intact cells were detected (Fig 6C). Urinary tubules were filled by stained particles and some cells were found in glomerulus (Fig 6D), but its integrity was disrupted.

## Discussion

The main findings of the present work was as follows:

1) Intravenous administration of ASCs significantly reduced the pulmonary microvascular hyperpermeability in sheep caused by cotton smoke inhalation. This was evidenced by significantly attenuated increases in lung lymph flow, an index of pulmonary microvascular fluid flux, pulmonary microvascular hyperpermeability index and reduced lung water content. These observations were supported by the findings that ASCs significantly reduced the systemic fluid retention, increased the plasma protein, prevented protein loss in lymph, and increased the urinary output. 2) The administration of ASCs significantly improved pulmonary gas exchange, evaluated by determining PaO_2_/FiO_2_ ratio (arterial/inspired air oxygen partial pressures), and pulmonary oxygenation index. The treatment also significantly reduced elevated airway pressures. The data from present study suggest that ASCs treatment delayed the onset of mild ARDS and prevented the development of moderate and severe ARDS as the PaO_2_/FiO_2_ ratio stayed above 300 up to 30hrs and above 200 in the remaining time period. It is worth noting that the intravenous administration of ASCs was well tolerated, as there were not any negative hemodynamic changes observed, including pulmonary arterial pressure during the 30-minute infusion time.

MSCs are multipotent cells with low immunogenicity that secrete multiple anti-inflammatory cytokines capable of modulating the immune response, attenuating bacterial infection by secretion of antimicrobial peptides, and controlling oxidative stress through the transfer of functional mitochondria to the damaged host cells [11, 19–21]. It has previously been shown that MSCs from different origins ameliorated the severity of acute lung injury [22–25]. In contrast to our research, a majority of these studies were conducted with the rodent model, and treated with bone marrow-derived human mesenchymal stem cells. The sheep model has proven to be an excellent paragon for biomedical research. The anatomy and physiology of the sheep respiratory system is well understood and much more comparable to humans. Hence, the ovine model is appropriate for vaccines, asthma pathogenesis, and inhalation treatment studies.

As mentioned, adipose-derived stem cells exerted a strong effect on systemic and pulmonary microvascular hyper-permeability caused by smoke inhalation; however, the study is limited by insufficiently described mechanistic aspects underlying these salutary effects. It has been shown that beneficial effects of MSCs are mediated by many different factors including their ability to modulate innate and adaptive immune cells, by enhancing anti-inflammatory pathways, such as IL-10 and IL-1 receptor antagonist [26, 27]. Previous studies reported that MSCs alleviate severity of organ injury by their antioxidative effects [28]. MSCs attenuate neutrophil activity and protect against ventilator-induced lung injury [29]. Pati S et al. reported that bone marrow derived mesenchymal stem cells inhibit inflammation and preserve vascular endothelial integrity in the lungs after hemorrhage shock by inhibiting leucocyte adhesion molecules [30]. In our previous studies, we have demonstrated important roles of oxidative stress, neutrophil activation, adhesion molecules, and pro-inflammatory cytokines, such as IL-1 and IL-6 in smoke inhalation-induced acute lung injury in sheep model [31–38]. Thus, based on previous studies by others and our group, we speculate that ASCs may have attenuated severity of smoke inhalation-induced acute lung injury by exerting their potent anti-inflammatory (secretory function) properties.

Previous studies also reported that MSCs attenuate lung injury through lipoxin A4 (LXA4) [39, 40]. It has also been shown that potent permeability factor angiopoietin-2 (ANG2) to play a critical role in the pathophysiology of ARDS [41–43]. We have measured both LXA4 and ANG2 in the lung tissue at 48 hours (time of necropsy); however, these values were not affected by ASCs treatment, suggesting that LXA4 and ANG2 pathways are not involved in the beneficial effect of ASCs on microvascular hyper-permeability and lung tissue injury at least at 48 hours.

Another limitation of our present study is that we were not able to measure inflammatory or permeability markers in the lung tissue at different time points after the injury. It is possible that the peak time of the expression of ANG2 and LXA4 occurred much earlier; as we have reported in our previous studies that inflammatory mediators (lung IL-1β, TNF-α, IL-6) in mice lung tissue peaked around 9 hours after the injury [36]. We have also reported on the time course of cytokines in sheep sepsis study, in which IL-6 and PARP activation in lung tissue peaked at 8 hours and 12 hours after injury [37].

In the present study, we have determined the distribution of intravenously administered ASCs. We found many structurally intact ASCs in the lung parenchyma 24 hours after the injury (microscopic image). Some intact cells were also spotted in the liver sinus. However, we were not able to find intact ASCs in spleen or urine in spite of the positive signals of green fluorescent protein (GFP) by whole organ imaging or flow cytometer.

Microscopically, we found numerous small particles stained with anti-GFP antibody in spleen and renal tubules. It is worth nothing that whole organ imaging gives some idea of cell distribution, but this method does not provide information on the cell morphology, while microscopic assay (immunohistochemistry) enables the determination of cell integrity.

Nevertheless, the above results suggest that most of the intravenously administered ASCs are deposited in the lung tissue, migrating to interstitial space. Currently, we have no evidence confirming their differentiation to lung epithelial or endothelial cells; however we do not exclude the possibility of their engraftment, thus repairing the injured cells—this hypothesis should be further investigated. It appears that there was none or minimal (liver) distribution of these cells in non-pulmonary organs at least 24 h injection (another limitation of this study is that were not able to harvest lung tissue at different times after the ASCs infusion).

Previously, numbers of investigators reported in vivo cell distribution. Schrepfer et al. demonstrated that most of intravenously (IV) infused MSCs were trapped in the lung 5 minutes after injection, and lung passing was size dependent [44]. Fischer et al. investigated the pulmonary first-pass effect and found MSCs in carotid artery immediately post- injection, but the numbers were less than 1% of infused cells [45]. Barbash et al. showed that IV injected MSCs were redistributed in liver, kidney and bladder 4 hours after injection using whole body fluorescent imaging [46]. These studies suggest that a small amount of cells pass through the lung microvasculature. However, this notion should be confirmed by future studies with frequent sampling at various time points after the infusion.

Nevertheless, we are reporting for the first time, to our knowledge, the beneficial effects of ASCs in a clinically relevant translation ovine model of acute lung injury induced by smoke inhalation. This is of a particular importance, because there are no clinical studies demonstrating efficacy of MSCs in ARDS patients expect two phase-1 safety studies with limited numbers of patients [12, 13]. Previously, our group demonstrated beneficial effects of clinical grade human bone marrow-derived MSCs in ovine model of ALI/ARDS induced by pneumonia sepsis [14].

In conclusion, the results of our previous and present studies as well as prior studies by others, strongly suggest that ASCs could be a safe and efficient therapeutic option for the treatment of ARDS patients. These studies also indicate a need for large, multicenter and prospective clinical studies on the safety and efficacy of ASCs in ARDS patients. Future studies exploring mechanistic aspects specifically focusing on the salutary effects of ASCs on endothelial permeability are warranted.

## Supporting information

S1 Data Data of control sheep; N = 5. (XLSX)

S2 Data Data of treatment sheep; N = 5. (XLSX)
